# The effects of chronic unpredicted mild stress on maternal negative emotions and gut microbiota and metabolites in pregnant rats

**DOI:** 10.7717/peerj.15113

**Published:** 2023-04-17

**Authors:** Rui Wang, Feng Zhao, Ye Li, Jiashu Zhu, Yifei Liu, Jiaqi Li, Guixiang Yao, Hongya Liu, Suzhen Guan, Shuqin Ma

**Affiliations:** 1 Ningxia Medical University, Ningxia, People’s Republic of China; 2 Key Laboratory of Environmental Factors and Chronic Disease Control, Ningxia, People’s Republic of China; 3 Chongqing Medical University, Chongqing, People’s Republic of China; 4 General hospital of Ningxia Medical University, Ningxia, People’s Republic of China

**Keywords:** Chronic stress during pregnancy, Negative emotion, Gut microbiota, 16S rRNA gene sequencing, Metabolomics

## Abstract

**Background:**

Chronic long-term stress is associated with a range of disorders, including depression and a variety of other chronic illnesses. It is well known that maternal exposure to psychosocial stress during pregnancy significantly increases the likelihood of adverse pregnancy outcomes. The gut microbiota has been a popular topic, it is a key mediator of the gut-brain axis and plays an important role in human health; changes in the gut microbiota have been related to chronic stress-induced health impairment, however, the relationship between maternal negative emotions and abnormal gut microbiota and its metabolites during maternal exposure to chronic stress during pregnancy remains unclear.

**Methods:**

Pregnant rats were subjected to chronic unpredicted mild stress (CUMS) to establish the rat model of chronic stress during pregnancy. The behavioral changes were recorded using sucrose preference test (SPT) and open-field test (OFT), plasma corticosterone levels were determined by radioimmunoassay, and a comprehensive method combining 16S rRNA gene sequencing and gas chromatography-mass spectrometry (GC-MS) metabolomics was used to study the effects of stress during pregnancy on the function of intestinal microbiota and its metabolites.

**Results:**

Chronic stress during pregnancy not only increased maternal plasma corticosterone (*P* < 0.05), but also caused maternal depression-like behaviors (*P* < 0.05). Chronic stress during pregnancy changed the species composition at the family level of maternal gut microbiota, the species abundance of *Ruminococcaceae* in the stress group (23.45%) was lower than the control group (32.67%) and the species abundance of *Prevotellaceae* in the stress group (10.45%) was higher than the control group (0.03%) (*P* < 0.05). Vertical locomotion and 1% sucrose preference percentage in pregnant rats were negatively correlated with Prevotellaceae (*r* =  − 0.90, *P* < 0.05). Principal component analysis with partial least squares discriminant analysis showed that the integration points of metabolic components in the stress and control groups were completely separated, indicating that there were significant differences in the metabolic patterns of the two groups, and there were seven endogenous metabolites that differed (*P* < 0.05).

**Conclusions:**

The negative emotional behaviors that occur in pregnant rats as a result of prenatal chronic stress may be associated with alterations in the gut microbiota and its metabolites. These findings provide a basis for future targeted metabolomics and gut flora studies on the effects of chronic stress during pregnancy on gut flora.

## Introduction

The body’s reaction to a number of harmful external causes is stress, which is a condition of physical and mental strain (Aderibigbe, Gureje & Omigbodun, 1993). Long-term stress may be detrimental to the body and major negative effects on health. Pregnant women perceive psychological stress under numerous acute and chronic strains throughout this specific stage of their pregnancy since they are filled with expectations and uncertainties about their unborn child (Shishehgar et al., 2014). In many studies, there is a significant association between perceived stress and its physical and psychological symptoms (Hjelm et al., 2017; Li et al., 2020). Severe chronic stress associated with pregnancy has a significant role in the emergence of anxiety and depression symptoms in pregnant women (Shangguan et al., 2021). Anxiety during pregnancy can be harmful to both the mother’s and the unborn child’s health.

The gastrointestinal tract, which is the largest immunological and metabolic organ in humans, comprises a range of intestinal flora that are thought to be crucial participants in the coordination of host physiology and disease (Qin et al., 2010). A number of factors, including nutrition, seasonality, lifestyle, stress, usage of antibiotics, or illness, can have an impact on intestinal flora (Li et al., 2021). Intestinal flora is a crucial element of human microecology and plays a crucial role in human immunity, metabolic equilibrium, and the prevention of pathogenic invasion, according to recent studies (Glenwright et al., 2017; Khosravi et al., 2014; Pickard et al., 2017). Especially for the special stage of female pregnancy, it plays a vital role (Nuriel-Ohayon, Neuman & Koren, 2016; Rautava et al., 2012). The ability to absorb nutrients to fulfill the nutritional and metabolic demands of pregnancy and lactation are made easier as a result of the dynamic remodeling that the maternal gut microbiota experiences throughout pregnancy, according to prior research (Koren et al., 2012). Indeed, some evidence suggests that the gut microbiota interacts with diet, medications, and stress, and that these exogenous factors may also influence the dynamics of microbiota composition that occur during pregnancy (Dawson et al., 2019; Karl et al., 2017; Rackers et al., 2018). The ratio between species falls out of balance when the balance of intestinal flora is disturbed by changes in the host or external environment, resulting in fewer good bacteria and more dangerous bacteria in the gut and negative symptoms in the organism.

The gut microbiota has regulatory effects on anxiety, mood, cognition, and pain, and this effect is mediated through the gut-brain axis. Recent research has demonstrated that being under stress changes the gut microbiota, which is linked to the emergence of social anxiety, depression, and stress-related behavior (Gur et al., 2017; Park et al., 2021; Tan et al., 2021). The gut-brain axis is a bidirectional regulatory axis between the brain and the gastrointestinal tract, which mainly includes the central nervous system, the autonomic nervous system, the enteric nervous system, the HPA axis and other structures, and each part is coordinated with others (Aziz & Thompson, 1998). As gastrointestinal discomfort is frequently accompanied by a negative emotional reaction, which in turn activates the central nervous system and causes additional alterations in gastrointestinal output, there is an interaction between emotions and the digestive tract (Person & Keefer, 2021). Similarly, neural transmission signals from the brain can change the gastrointestinal tract’s muscular, sensory, and secretory processes, affecting microbial equilibrium (Clarke et al., 2014; Rajanala, Kumar & Chamallamudi, 2021).

Research shows that under the condition of chronic stress, the beneficial bacteria in the gastrointestinal tract of rats will decrease and the intestinal flora will be out of balance (Bailey & Coe, 1999). Older pregnant women have increased the OTUs of *F. prausnitzii* and *B. obeum* in their gut microecosystems but decreased OTUs in *P. copri* compared to younger pregnant women (Yang et al., 2020). Another study has revealed that the relative abundance of *Streptococcaceae* elevate in patients with major depression (Barandouzi et al., 2020). In conclusion, there is a link between intestinal flora imbalance and brain-gut axis dysfunction, however it is unclear if alterations in intestinal flora are related to chronic stress-induced brain-gut axis dysfunction during pregnancy. Therefore, we built an animal model by producing chronic unpredictable mild stress (CUMS) throughout pregnancy in order to investigate the effects of maternal experience of chronic stress during pregnancy on the intestinal flora and its metabolites, starting from maternal stress.

## Materials and Methods

### Animals

Thirty-eight adult Sprague-Dawley (SD) rats with specific pathogen free (SPF) were selected and purchased from the Experimental Animal Center of Ningxia Medical University, with the certificate number: SCXK (Ning) 2015-0001. A total of 16 female rats (body weight 200 ± 20 g) were divided randomly into stress model group and control group, with eight rats in each group. The stress group was reared in a single cage, and the control group was reared with four animals per cage. 12 male rats (body weight 230 ± 20 g) were randomly divided into control stress model mating group (eight rats) and mating group (four rats). The researchers knew all about the groups. The male rats were housed in standard cages (four animals in each cage), with free access to food and water. The whole process of animal experiments was carried out in a common animal room. The temperature of the breeding environment was 18∼20 °C, and the relative humidity was 50%∼60%, to ensure adequate fresh and dry feed and free drinking water. The care and use of all animals in this study were approved by the Laboratory Animal Welfare Ethical Review Committee of the Laboratory Animal Center of Ningxia Medical University (Number: IACUC-NYLAC-2019-089). Rats were anesthetized using 0.3 ml of 10% chloral hydrate per 100 g of body weight. Because this study investigated the effects of chronic stress stimulation during pregnancy on rats, if female rats were not pregnant during the study period, they were eliminated and given excessive anesthesia for euthanasia to shorten the time of death and reduce the suffering of the experimental animals. Finally, our experiments mainly study the SD rats during pregnancy. When the SD rats were produced, both the mother and the offspring were used in other experiments of this group.

### Methods

#### Procedure of chronic unpredictable mild stress (CUMS) exposure

**Figure 1:**
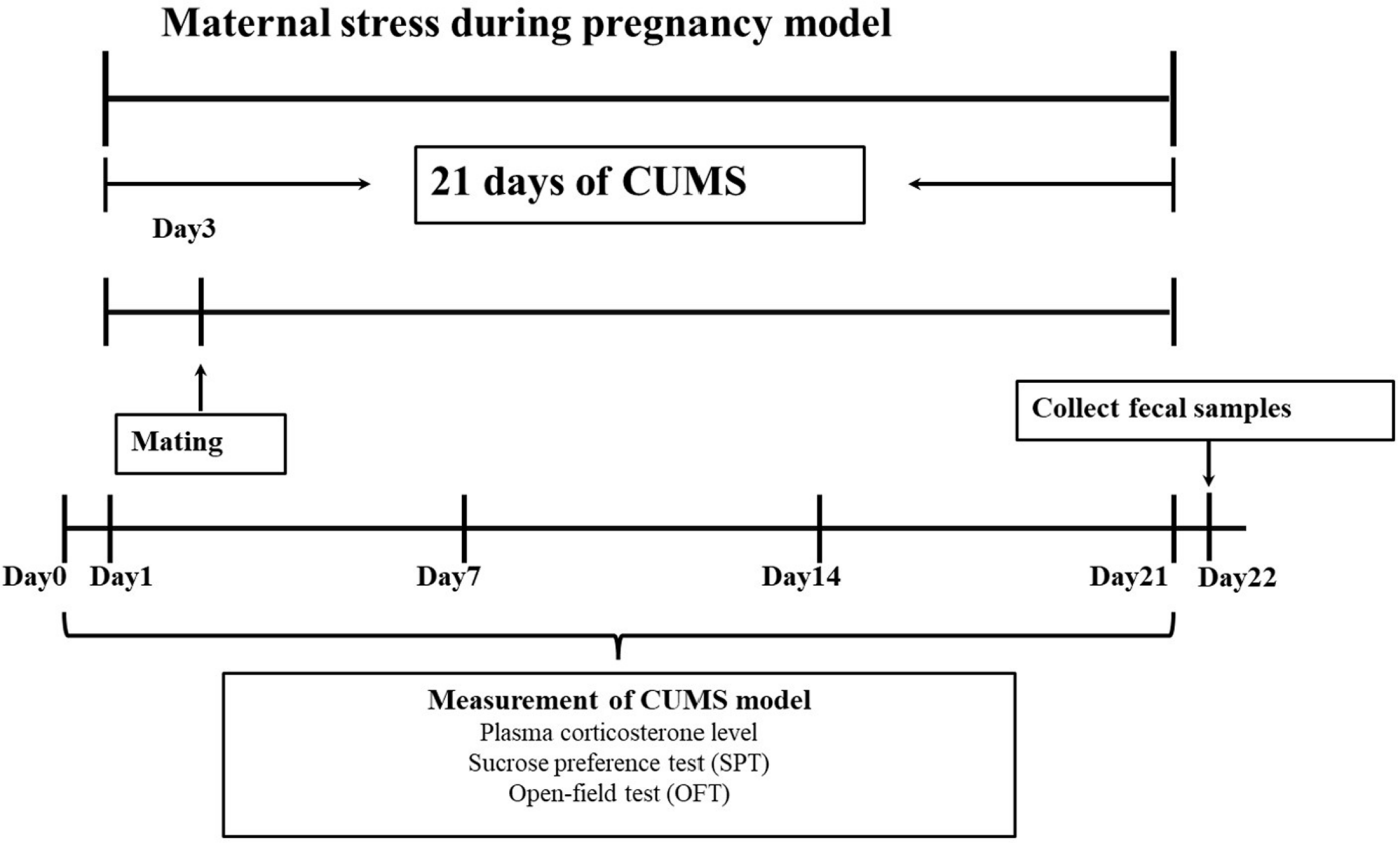
Experimental protocol. In this study, a pregnant animal model was created using chronic unpredictable mild stress (CUMS). There were 21 days in all. An open-ended test (OFT), a sucrose preference test (SPT), and intraocular venous blood collection were performed on female rats 1 day before and 1, 7, 14 and 21 days after stress. Fresh faeces were collected from female rats at the end of stress.

After a week of adaptive feeding, chronic unpredictable mild stress (CUMS) was using to establish an animal model (Guan et al., 2016). Specific stress methods included: (1) 24 h food deprivation; (2) 24 h crowded environment (8 cages, cage tilted 30° ); (3) 24 h water deprivation; (4) 24 h humid environment (humidity 60%–70%); (5) 1 h warm water swimming (31 °C); (6) 30 min shaking (1 time ⋅ s-1); (7) 30 min behavior restraint; (8) 5 min heat stress (42 °C); (9) 1 min tail clamping (one cm from the tip of the tail). One of the nine different stressors was randomly administered each day during the model period of 21 consecutive days, meanwhile, stress stimulation was given in another feeding room with the same environmental conditions (the light intensity and temperature of the two rooms were basically the same). After the stress was completed, the rats were sent back to the original feeding room. The entire experimental flow was shown in Fig. 1.

#### Mating

On the third day of the CUMS cycle, cage mating was carried out (Guan et al., 2016). The female rats in the stress model group and the male rats in the stress model mating group were mated in cages according to a ratio of 1:1. The female rats in the control group mated with the male rats in the control mating group were mated in cages at a ratio of 2:1. Because the vaginal suppository of rats is easy to fall off, the pregnancy was determined by observing the vaginal suppository and examining sperm in vaginal smears. After the female rats were confirmed to be pregnant, the male and female rats were separated, and the control group rats were reared with two rats per cage (one rat per cage after GD18), while the stress model group rats were reared alone (one rat per cage). Stress stimulation did not interrupt mating.

### Confirmation of chronic stress during pregnancy through measurement of plasma corticosterone level

The medial canthus vein was utilized by all female rats to draw blood on the baseline (day before stress), the first, seventh, and fourteenth days, as well as to collect plasma. At around 2:00 p.m., female rat’s plasma was taken after each behavioral test. The level of plasma corticosterone was measured using plasma. The obtained plasma was kept at −80 °C after being centrifuged at 3,000 rpm for 20 min at 4 °C with blood samples. According to the manufacturer’s recommendations, corticosterone was tested with a radioimmunoassay (RIA) kit (Coat-A-Count, Diagnostics Products Corporation). The RIA’s within-group variance varied between 3.2% and 4.7%. Using the conversion procedure shown below, the plasma corticosterone levels were calculated from the cortisol values: Cortisol level minus 50 equals corticosterone level (Liu et al., 2004).

### Determination of emotional behavior changes of pregnant rat

#### Sucrose preference test (SPT) (Iñiguez et al., 2009)

The experiment was preceded by adaptation training to allow female rats to adapt to sugary drinking water by placing 2 vials containing 1% sucrose water in each cage for 24 h. The female rats in each group were subjected to a sugar-water preference test on day 1 before stress, day 7, 14 and 21, while 2 vials of weighed water (one vial of 1% sucrose water and one vial of pure water) were given to each rat in each cage, and after 0.5 h, the positions of the two vials were exchanged, and after 0.5 h, the two vials were removed and measured. Food and water were fasted for 24 h before the experiment. After 30 min, the two water bottles’ right and left placements were switched at random among the several animals. Pure water intake, sugar water consumption, total liquid consumption, and a 1% preference for sucrose were all noted. Consumption of sugar water divided by total fluid consumption equals 100% for a 1% sucrose preference percentage.

#### Open-field test (OFT) (Hu et al., 2017)

General motor activity and anxiety-like behavior were assessed by open-field test (OFT) in adult rats. The OFT was measured in all female rats on day 1 before stress and the 1st, 7th, 14th and 21st days after stress. Each rat was gently placed in the center of the test area and observed for 3 min. An opaque laboratory box (80 × 80 × 40 cm) was divided into 25 squares of equal size and the surrounding walls were painted black. The rats were placed in the central square and the number of squares the rats walked across in 3 min was recorded (only squares where the rats landed on all fours were recorded as the horizontal activity score) and the number of times the rats stood (the number of times the rats climbed the wall or took off with their front paws was recorded as the vertical score). Each rat was measured once for 3 min. After simultaneous observation by two observers, one score was observed by each observer and finally the average of the two observations was taken. After each test, the test box was cleaned with 10% ethanol.

### Determination of maternal gut microbiome using 16S rRNA gene sequencing

By obtaining fecal samples from six randomly chosen rats in each group and 16S rRNA gene sequencing them the following morning, after the stress cycle has ended, it was possible to compare the differences in the gut microbial communities between the Control and Stress groups. With the use of the E.Z.N.A. Stool DNA kit, total DNA was extracted (Omega Bio-Tek, Norcross, GA, USA). Using the following primers, the bacterial 16S rRNA gene’s V3-V4 region was amplified by polymerase chain reaction (PCR): 338F 5′-ACTCCTACGGGAGGCAGCA-3′and 806R 5′-GGACTACHVGGGTWTC TAAT-3′. 95 °C for 3 min, then 27 cycles of 95 °C for 30 s, 55 °C for 30 s, and 72 °C for 45 s, with a final step of 72 °C for 10 min, were the PCR conditions. Amplification was verified using electrophoresis on 2% agarose gel. The AxyPrep DNA kit (AXYGEN, Tewksbury, MA, USA) was used to purify the PCR results before they were sequenced on the Illumina HiSeq platform. In order to regulate and filter the sequence quality, the PE reads generated by MiSeq sequencing were first spliced in accordance with the overlap relationship. Following sample separation, a community bar plot analysis was carried out at the family level. *β*-Diversity was assessed using the Bray-Curtis distance technique and visualized using main coordinate analysis (PCoA). Function was predicted and information on the COG family corresponding to operational taxonomic units was obtained using the Phylogenetic Investigation of Communities by Reconstruction of Unobserved States (PICRUSt) method (OUT). A functional abundance spectrum was obtained by searching the egg NOG database for each COG’s description and function. The Majorbio I-Sanger Cloud Platform, a free online service, was used to evaluate the data (http://www.i-sanger.com).

### Determination of maternal metabolites using gas chromatography-mass spectrometry (GC-MS) metabolomics

#### Sample processing for metabolomics

A total of 50 mg of feces sample was precisely weighed into a 2 ml centrifuge tube and 200 µL of chloroform, 0.5 ml of a methanol-water solution (CH_3_OH: H_2_O v:v = 4:1, containing 0.02 mg/mL internal standard L-2-chloro-phenylalanine) was added and ground for 3 min of 50 Hz grind, and 30 min of ultrasonic extract. Following standing at −20 °C for 30 min, 500 µL of the supernatant was centrifuged at a low speed for 15 min (13000 rcf, 4 °C), transferred in a glass derivatization container, and nitrogen-blown dry; in a shaking incubator, 80 liters of a 15 mg/mL solution of methoxyamine hydrochloride and pyridine hydrochloride was added, vortexed and shaken 2 min, and then oximize for 90 min at 37 °C; after that, 80 µL of BSTFA derivatization reagent (containing 1% TMCS) was added, followed by a 2-minute vortex and a 60-minute reaction at 70 °C; the sample was removed and let sit at room temperature for 30 min to get it ready for GC/MS metabolomics analysis.

#### Metabolomics profiling

The 8890B-5977B gas chromatography-mass spectrometer from Agilent is the analytical device used in this investigation (Agilent, Santa Clara, CA, USA). The material was put into the GC-MS apparatus for split mode analysis following derivatization. A quality control sample (QC) was created during the experiment in order to assess the stability of the analysis system during the computer process. The HP-5MS UI receives the sample. It entered mass spectrometry detection after separation on a capillary column (30 m × 0.25 mm × 0.25 µm, Agilent J&W Scientific, Agilent 19091S-433). Mass Hunter Workstation Quantitative Analysis (v10.0.707.0) software preprocessed the initial GC/MS data when the computer finished in order to provide the final data matrix for further analysis. The Fiehn public database served as the primary resource for the simultaneous search and identification of the metabolites.

#### Data processing of metabolomics

Agilent Technologies’ MassHunter Workstation software was used to convert data that had been gathered in Agilent, d format to mzXML. When data were filtered by intensity, only signals with intensities larger than 1,000 were taken into account. The data was transformed and processed using XCMS Online for peak selecting, alignment, integration, and peak intensity extraction. A two-tailed Welch’s *t*-test (*P* 0.05) was performed to characterize individual metabolite changes between control and stress groups. With a 10-ppm mass accuracy criteria, the Human Metabolome Database (HMDB; http://www.hmdb.ca/), METLIN (http://metlin.scripps.edu), and Kyoto Encyclopedia of Genes and Genomes (KEGG) databases were searched for the precise masses of molecular characteristics with significant alterations. To identify the metabolites, GC/MS data were generated using the stored matched precise masses.

### Statistical analysis of data

The Statistical Package for the Social Sciences (version 23.0) was used for all statistical analyses, expressed as mean ±  SD, and GraphPad Prism was used to create the graphics (version 5.0). Two-way repeated measurements analysis of variance was used to assess differences between the means of maternal data for the plasma corticosterone level, open-field test, and sucrose preference test. Student’s *t* test was using to make multiple comparisons at the different times. Statistics were judged significant at *P* < 0.05.

## Results

### Effects of CUMS on pregnant rat

#### CUMS increased plasma corticosterone levels of pregnant rat

**Figure 2:**
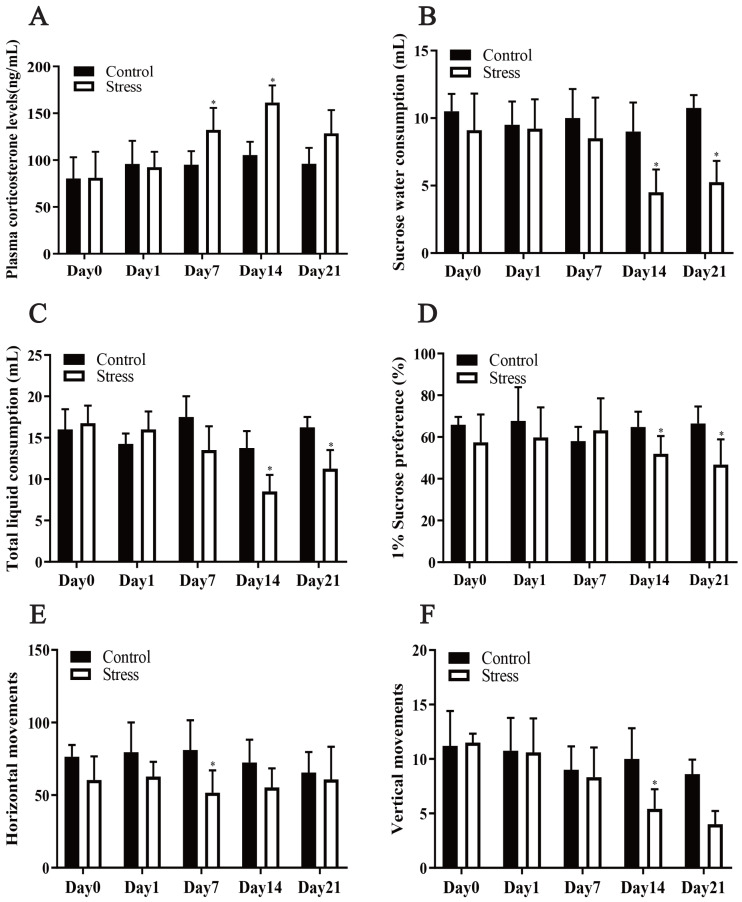
Effect of CUMS model on mantel. (A) plasma corticosterone levels; (B–D) SPT; (E–F) OFT. Data are expressed as the mean ± standard deviation (SD) (*n* = 8 per group); ^∗^compared with the control group on the same time point, *P* < 0.05.

Chronic stress significantly affected the plasma corticosterone levels of the female rats, according to the repeated measurement analysis of variance (*F* = 7.717, *P* = 0.024). Stress significantly altered the corticosterone level of the stress model group (*F* = 6.076, *P* = 0.01). Additionally, a t test showed that after being exposed to stress for 7 days, the plasma corticosterone level of the stress group was greater than that of the control group (*t* = 2.341, *P* = 0.047). Following exposure to stress for 14 days, the plasma corticosterone level of the Stress group increased to its highest value and was greater than that of the Control group (*t* = 5.414, *P* = 0.001), showing that the Stress group was in a chronically stressed condition (Fig. 2A).

#### CUMS decreased maternal liquid consumption

The repeated measurement analysis of variance revealed that chronic stress significantly affected sugar water consumption, total liquid consumption and 1% sucrose preference in the Stress rats (*F* = 18.100, 9.007, 5.528; *P* = 0.002, 0.013, 0.041). It showed that stress affected fluid consumption index of rats during pregnancy. Following exposure to stress for 14 and 21 days, it was manifested that sucrose-intake of the Stress rats reduced compared with Control rats by *t* test (*t* = 2.557, 2.897; *P* = 0.029,0.016) (Figs. 2B–2D).

#### CUMS reduced maternal horizontal and vertical movements

The horizontal and vertical movement scores of the Stress group and Control group were compared, and the differences between them were statistically significant (*F* = 36.339, 5.680; *P* = 0.001,0.049). The results of *t* test analysis indicated that the horizontal movement of the model group was less than that of the control group after 7 days of exposure to stress (*t* = 2.950, *P* = 0.013) (Fig. 2E), and after exposure to stress for 4 days, vertical movements in the stress group were also fewer (*t* = 3.257; *P* = 0.005) (Fig. 2F). Pregnant mice in the stress group showed reduced voluntary exploratory behaviour in the new environment, were less able to adapt to the unfamiliar environment, were less excitable, had significantly lower cognitive abilities and were in a state of anxiety compared to the control group.

### CUMS changed the maternal gut microbiota

#### CUMS reduced the diversity and abundance of maternal gut microbiota

**Figure 3:**
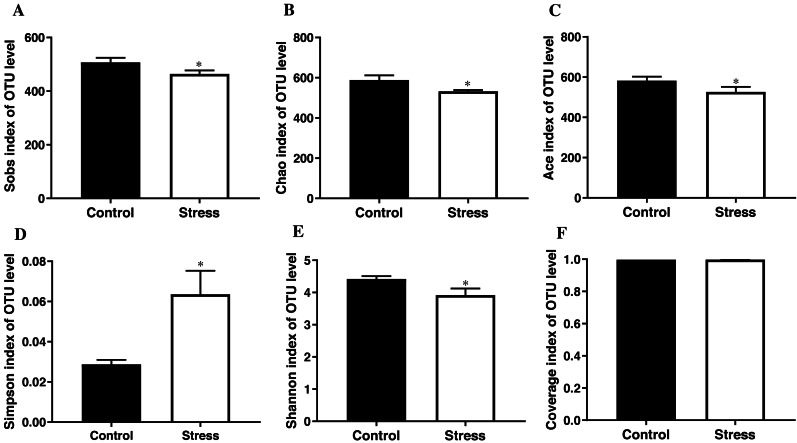
*α*-diversity indices of gut microbiota in pregnant rat. (A) OTU number of gut microbiota; (B–C) abundance of gut microbiota; (D–E) diversity of gut microbiota; (F) coverage of gut microbiota. Data are expressed as the mean ± SD (*n* = 6 per group); ^∗^ compared with the control group, *P* < 0.05.

The student’s *t* test results documented that the Sobs index, Chao index, Ace index, and Shannon index of the control group were higher than those of the stress group (*t* = 3.419, 3.626, 3.706, 3.900; all *P* < 0.05); the control group’s Simpson index was lower than the stress group (*t* =  − 3.890, *P* < 0.05); Converge index had no difference between the two groups (*P* > 0.05) (Fig. 3), indicating that CUMS significantly reduces the species richness and diversity of the maternal gut microbiota.

#### CUMS changed the species composition at the family level of maternal gut microbiota

**Figure 4:**
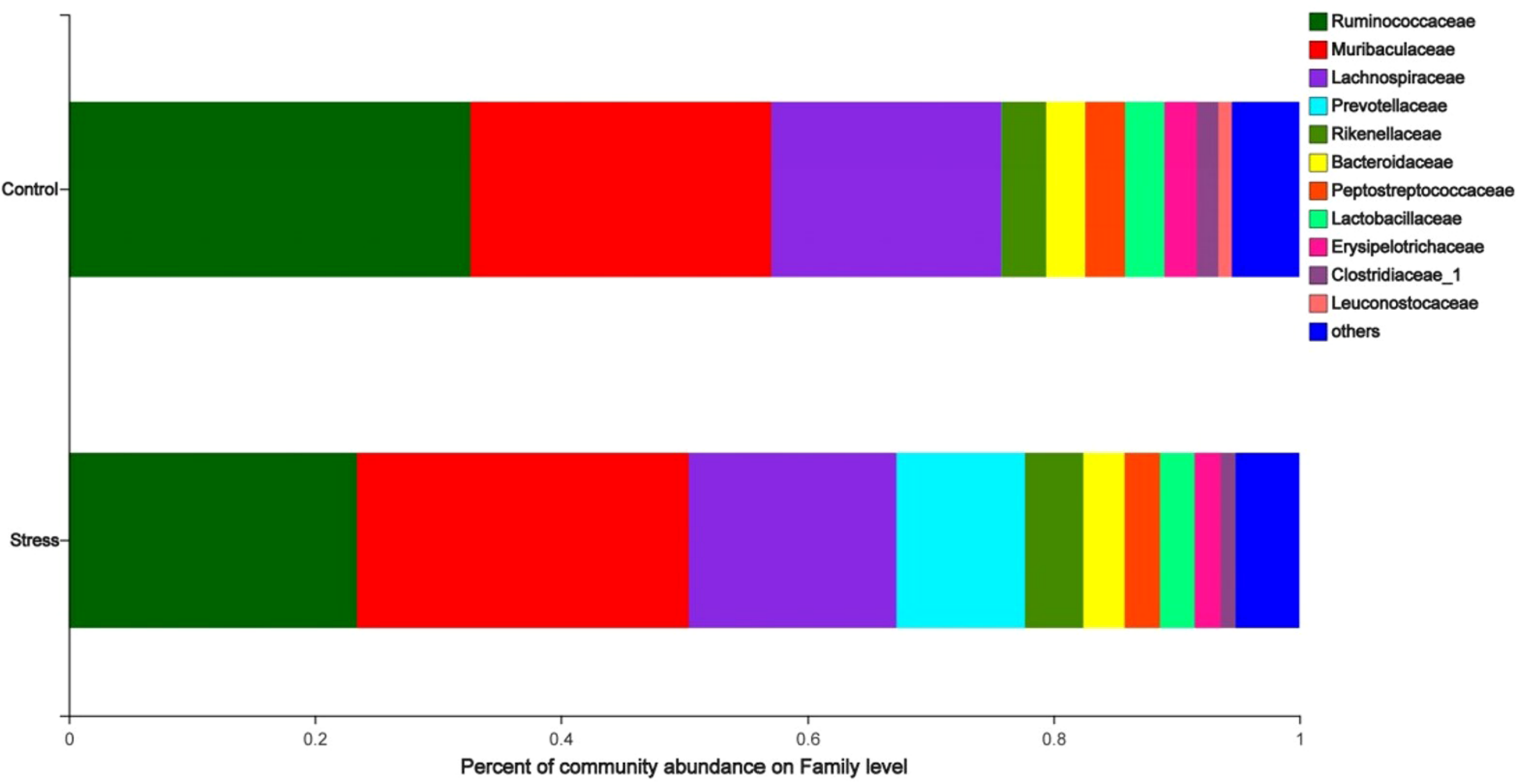
Family level analysis of gut microbiota in maternal gut microbiota. The ordinate was the name of the group and the abscissa was the proportion of species in the group. The pillars of different colored represent different species and the length of the pillars represented the proportion of the species.

The analysis of the species composition of the maternal gut microbiota family level shows that the relatively high species are *Ruminococcaceae*, *Muribaculaceae*, *Lachnospiraceae*, *Prevotellaceae*, and *Rikenellaceae*. The species abundance of *Ruminococcaceae* in the stress group (23.45%) was lower than the control group (32.67%); the species abundance of *Prevotellaceae* in the stress group (10.45%) was higher than the control group (0.03%) (*P* < 0.05) (Fig. 4), indicated that CUMS changes the species composition at the family level of maternal gut microbiota.

### The effects of CUMS on maternal fecal metabolism biomarkers

#### Metabolic phenotype analysis

**Figure 5:**
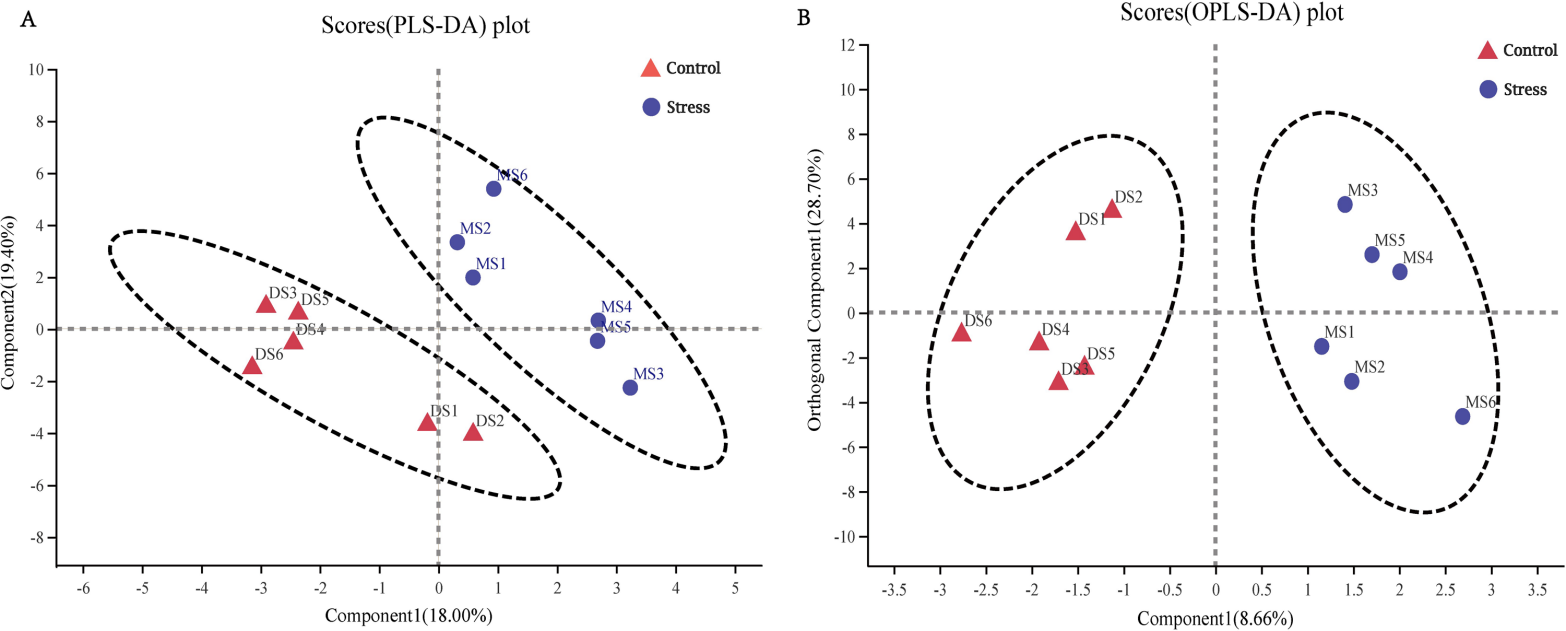
Metabolic phenotype analysis. (A) PLS-DA score chart, the greater the degree of separation between the two groups of samples in the figure, the more significant the classification effect. Component 1, first principal component interpretation degree (18.00%), Component 2, principal component interpretation degree (19.40%). (B) OPLS-DA score chart, Component 1 (8.66%), Orthogonal Component 1 (28.70%).

Variables with an important in projection (VIP) greater than one were considered differential variables in the PLS-DA analysis. The samples between the control group and the stress group could be separated well, and they were all within the confidence ellipse, and no abnormal points appeared, therefore, the sample data was reliable, and the difference in metabolites between the control group and the stress group can be preliminarily judged (Fig. 5A). OPLS-DA analysis showed that the samples of the control group and the stress group could be better separated, and it could be judged that the metabolites in the feces of the stress group had changed significantly (Fig. 5A).

#### VIP value analysis and KEGG compound classification

**Figure 6:**
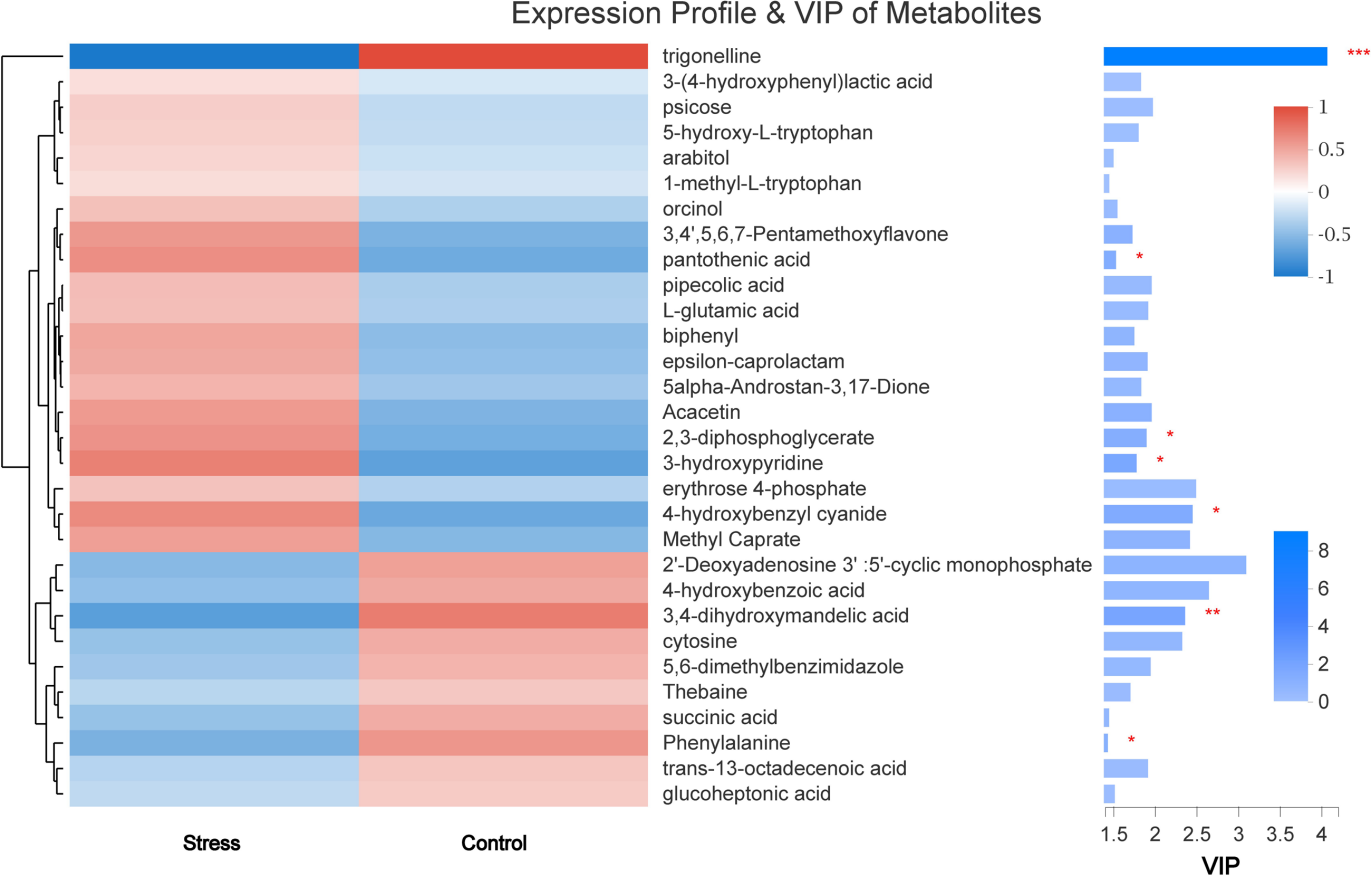
The metabolite clustering dendrogram is on the left, on the right is the VIP bar graph of metabolites. The length of the bar represents the contribution value of the metabolite to the difference between the two groups. ^∗^, *P* < 0.05.

The VIP value obtained in OPLS-DA was used to screen out differences in metabolites between groups (VIP > 1 and *P* < 0.05). Finally, seven kinds of small molecular metabolites with biological significance were obtained. Among them, the expressions of *trigonelline*, *3,4-dihydroxymandelic acid*, and *Phenylalanine* in the stress group were lower than those in the control group (all *P* < 0.05). The expression of *pantothenic acid*, *2,3-diphosphoglycerate*, *3-hydroxypyridine*, and *4-hydroxybenzyl cyanide* was significantly increased in the Stress group (all *P* < 0.05) (Fig. 6).

#### Metabolic pathway analysis

The metabolic set of the above-mentioned differential metabolites was established for pathway analysis. Compared with the control group, 11 metabolic pathways in the feces of rats in the stress group changed significantly (all *P* < 0.05) (Table 1). Nicotinate and nicotinamide metabolism, aminoacyl-tRNA biosynthesis, protein digestion and absorption, Cyanoamino acid metabolism, Central carbon metabolism in cancer, phenylalanine, tyrosine and tryptophan biosynthesis, beta-alanine metabolism, glycolysis/gluconeogenesis, mineral absorption, pantothenate and CoA biosynthesis, these pathways mainly involve protein biosynthesis, amino acid metabolism, sugar metabolism and intestinal microbial metabolism.

**Table 1:** Metabolic pathway analysis.

*Num*	Pathway description	Pathway_ID	*Ratio_in_pop*	*P*
1	Nicotinate and nicotinamide metabolism	map00760	55/5997	0.045
2	Aminoacyl-tRNA biosynthesis	map00970	52/5997	0.0426
3	Protein digestion and absorption	map04974	47/5997	0.0386
4	Vitamin digestion and absorption	map04977	39/5997	0.0321
5	Cyanoamino acid metabolism	map00460	45/5997	0.037
6	Central carbon metabolism in cancer	map05230	37/5997	0.0305
7	Phenylalanine, tyrosine and tryptophan biosynthesis	map00400	35/5997	0.0289
8	beta-Alanine metabolism	map00410	32/5997	0.0264
9	Glycolysis / Gluconeogenesis	map00010	31/5997	0.0256
10	Mineral absorption	map04978	29/5997	0.024
11	Pantothenate and CoA biosynthesis	map00770	28/5997	0.0231

### Correlation analysis of gut microbiota and emotional factors

**Figure 7:**
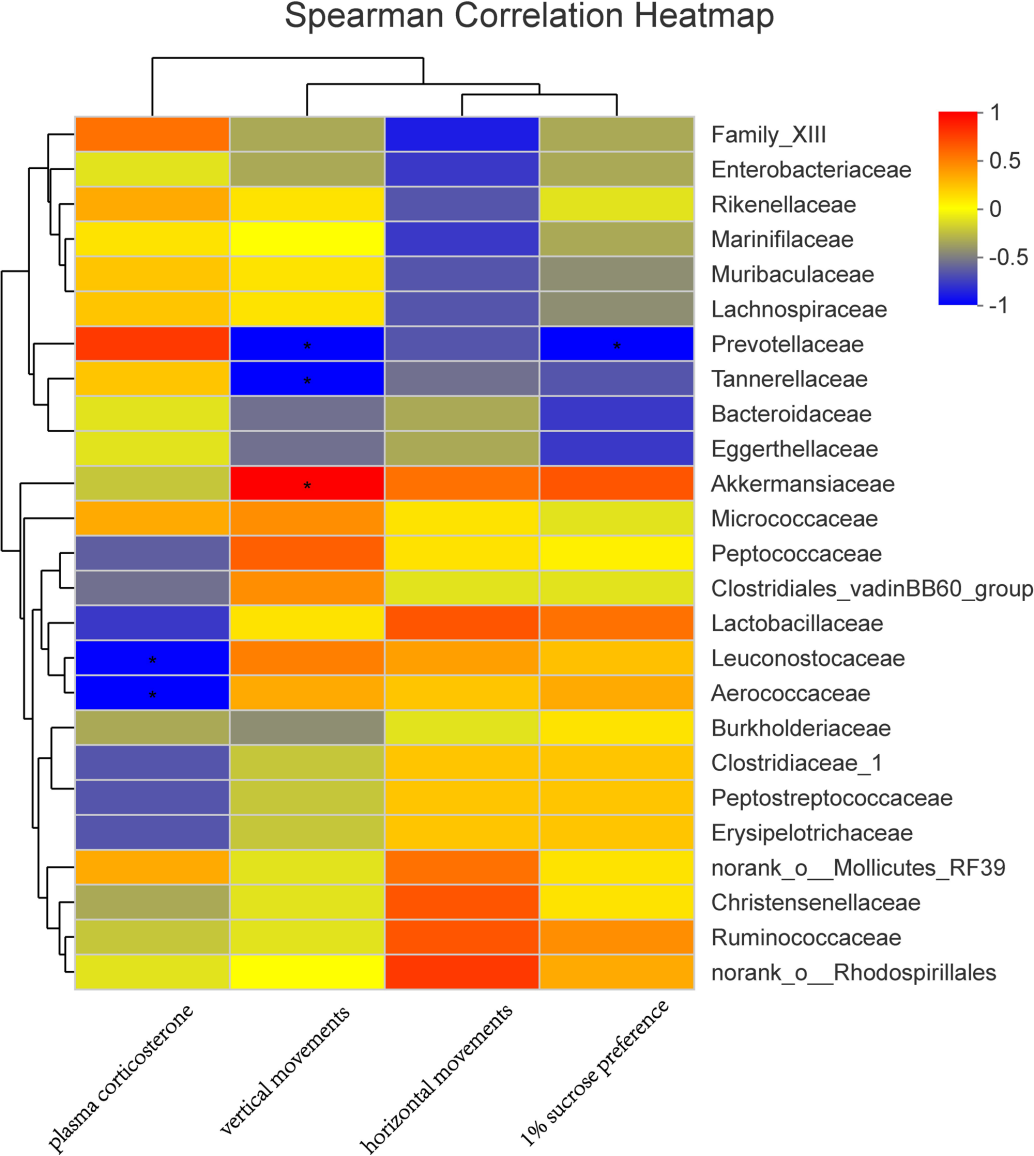
Correlation analysis between environmental factors and maternal gut microbiota species. The abscissa represents the environmental factor, the ordinate represents the species name, the legend on the right is the color interval of different *R* values; ^∗^ *P* < 0.05.

In a Spearman correlation analysis, the vertical movement was negatively correlated with *Prevotellaceae* (r =−0.90, *P* < 0.05), and the 1% sucrose preference percentage is negatively correlated with *Prevotellaceae* (r =−0.90, *P* < 0.05). Plasma corticosterone was also negatively correlated with *Leuconostocaceae* (r =−0.89, *P* < 0.05) and *Aerococcaceae* (r =−0.90, *P* < 0.05) (Fig. 7).

### Correlation between the gut microbiome and metabolites

**Figure 8:**
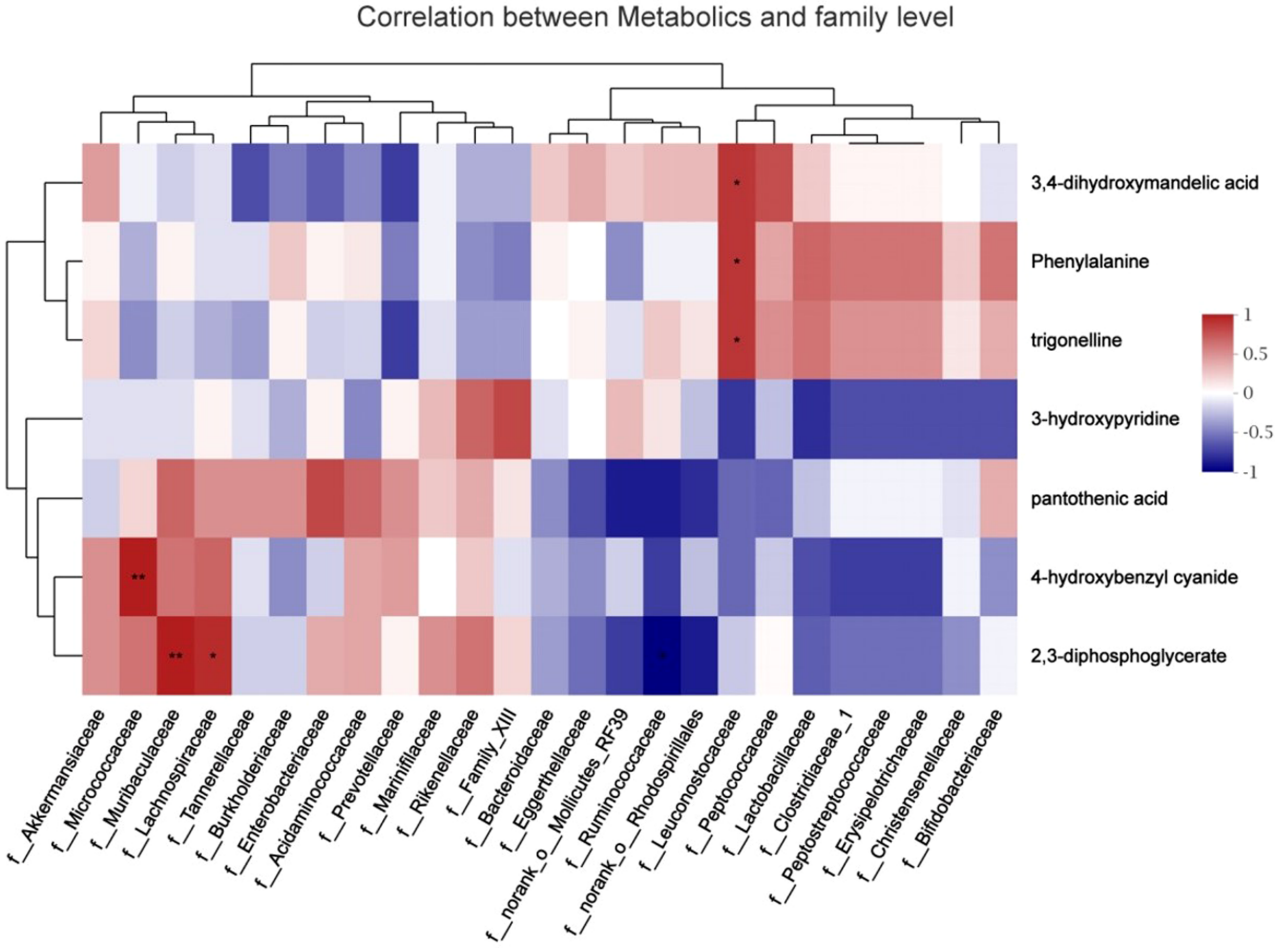
Correlation analysis between fecal metabolites and maternal gut microbiota species. The abscissa represented the species name, the ordinate represented the fecal metabolites, the legend on the right was the color interval of different *R* values; ^∗^ *P* <0.05.

To explore the functional correlation between the gut microbiome changes and metabolite perturbations, a correlation matrix was generated by calculating the Spearman’s correlation coefficient (Fig. 8). f_*Leuconostocaceae* was positively correlated with phenylalanine (*r* =0.85, *P* < 0.05), positively correlated with trigonelline (*r* =0.85, *P* < 0.05), and positively correlated with 3,4-dihydroxymandelic acid (*r* =0.85, *P* <  0.05); 2,3-diphosphoglycerate was negatively correlated with f__*Ruminococcaceae* (*r* =  − 0.89, *P* <  0.05); it was positively correlated with f__*Muribaculaceae* (*r* =0.94, *P* <  0.05); it was positively correlated with f__*Lachnospiraceae* (*r* =0.88, *P* <  0.05); f__*Micrococcaceae* and 4-hydroxybenzyl cyanide are positively correlated (*r* =0.94, *P* <  0.05). In summary, stress during pregnancy induced a significant taxonomic perturbation in the gut microbiome, which in turn substantially altered the metabolomic profile of the gut microbiome, as evidenced by changes of diverse gut microflora-related metabolites.

## Discussion

The chance of unfavorable pregnancy outcomes and mother cortisol levels are both considerably increased by exposure to certain psychosocial stresses or stressful physical activities during pregnancy, according to epidemiological studies (Lautarescu et al., 2020). The current investigation supports the conclusion that maternal in the chronic stress model group had plasma corticosterone levels that were greater than in the control group. However, plasma corticosterone levels declined on day 21 after stress stimulation, which may be connected to the HPA axis’s diminished expression of the final phases of stress adaption (De Prins et al., 2020). Overall, the rise in plasma corticosterone levels indicates that a successful rat model of persistent stress during pregnancy has been constructed. As evidenced by decreased mobility and exploration in the open field test and a preference for sugar water in the sucrose preference test, the findings, which are consistent with those of other studies, also showed that chronic stress during pregnancy increases the likelihood of unfavorable pregnancy outcomes (Han et al., 2018; Huang et al., 2019).

Studies have shown that an organism’s intestinal flora alters when it is under stress (Foster, Rinaman & Cryan, 2017). Intestinal flora in some patients with chronic and psychiatric illnesses frequently exhibits reduced alpha diversity (Cotillard et al., 2013; Kang et al., 2018; Karlsson et al., 2013; Prehn-Kristensen et al., 2018). Additionally, there is debate regarding the diversity of the gut microbiota in those who suffer from depression and anxiety disorders (Jiang et al., 2015; Kelly et al., 2016; Sanada et al., 2020; Simpson et al., 2020; Simpson et al., 2021). This study discovered that compared to controls, women who experienced chronic stress during pregnancy had considerably less alpha diversity of gut flora.

Chronic stress during pregnancy was found to have significantly lower alpha diversity of intestinal flora than the control group, indicating that anxiety during pregnancy can reduce alpha diversity. Jiang et al. (2015) indicated that the Shannon index of alpha diversity in depression was increased, an observation which was contrary in our study. Furthermore, Naseribafrouei et al. (2014) did not report change in alpha diversity. These results manifested that chronic stress during pregnancy can reduce the species abundance and diversity of the maternal gut microbial while changing.

The most prevalent bacteria at the family level were Ruminococcaceae, Muribaculaceae, Lachnospiraceae, Prevotellaceae, and Rikenellaceae. *Prevotaceae* species abundance was found to be considerably higher in the samples from the chronic stress during pregnancy group than in the samples from the control group, which was an intriguing finding in our study. These findings imply that persistent stress during pregnancy can change the variety and quantity of species in the gut microorganisms of the mother. Prevotella abundance was suggested a potential characteristic parameter for severe depression due to its favorable correlation with immunological characteristics (Gu et al., 2020).

Metabolomics, a methodical study approach, directly reflects alterations in the body’s metabolic processes and states (Zubeldia-Varela et al., 2020). One of the frequently utilized analysis techniques in metabonomics is the gas chromatography-mass spectrometry (GC-MS) method, which makes it easier to identify metabolites.

Our findings showed that the chronic stress during pregnancy group had considerably lower levels of trigonelline, 3,4-dihydroxymandelic acid, and phenylalanine than the control group. In addition, the stress group showed up-regulated expression of pantothenic acid, 2,3-diphosphoglycerate, 3-hydroxypyridine, and 4-hydroxybenzyl cyanide.

One of the necessary amino acids in the human body, phenylalanine works with tyrosine to create the neurotransmitters and hormones the body needs (Sánchez-López et al., 2016). It also plays a role in the metabolism of glucose and fat. Increased phenylalanine levels interfere with the production of tyrosine, which results in increased thyroxine levels and phenylketonuria (Peat & Garg, 2016; van Spronsen et al., 2017). According to Taskinen & Nikkilä (1979), excessive phenylalanine causes body glycometabolism, as well as fat and amino acid metabolic problems. Phenylalanine also activates pyruvate and enters the glycolytic cycle. The true attractant is 3,4-dihydroxymandelic acid, a norepinephrine metabolite produced by the activities of the bacterially encoded enzymes tyramine oxidase (Tyn A) and aromatic aldehyde dehydrogenase (ALDH) (Sule et al., 2017). Additionally, phenylalanine activates pyruvate and enters the glycolytic cycle. Excessive phenylalanine also causes metabolic abnormalities related to fat and amino acids as well as body glycometabolism (Taskinen & Nikkilä, 1979). The true attractant is 3,4-dihydroxymandelic acid, a norepinephrine metabolite produced by the activities of the bacterially encoded enzymes tyramine oxidase (Tyn A) and aromatic aldehyde dehydrogenase (ALDH) (Sule et al., 2017).

It plays a significant signaling role in the milieu of the gut. Niacin’s (vitamin B3 methylation) product, gogonelline, is a significant pyridine alkaloid whose antioxidative benefits on the brain have also been shown in models of various cognitive disorders (Farid et al., 2020). The molecular mechanisms of 3-hydroxypyridine breakdown in microbes have been investigated for a long time, although they are still unknown. In Actinobacteria, Rubrobacteria, Thermoleophila, and Alpha-, Beta-, and Gamma-proteobacteria, the 3-hydroxypyridine gene cluster was discovered to be common, and the genetic organization of the 3-hydroxypyridine dehydrogenase gene clusters in these bacteria exhibits high variety (Wang et al., 2020). Red blood cells get their energy from anaerobic gluconeogenesis, which also produces the metabolite 2,3-diphosphoglycerate, which, by binding to hemoglobin, encourages oxygen release (Järemo et al., 2019; Thomas et al., 1974). Red cells provide their energy by the anaerobic gluconeogenesis, which also generates the metabolite 2,3-diphosphoglycerate (Järemo et al., 2019), by binding to hemoglobin, the 2,3- diphosphoglycerate promotes oxygen release (Thomas et al., 1974). Metabolites were shown to engage 11 metabolic pathways, mostly those for amino acid, sugar, and gut microbial metabolism, according to KEGG pathway enrichment analysis. Some intestinal bacterial families were discovered to be strongly linked with changes in metabolites related to intestinal flora by correlation analysis.

The findings demonstrated that stress during pregnancy would result in substantial alterations in intestinal flora, which would then substantially affect the metabolic profile of the gut microbiome. The abundance of Leuconostocaceae decreased in the stress group compared to the control group as a result of stress, and a correlation analysis revealed a positive correlation between Leuconostocaceae and 2,3-diphosphoglycerate, resulting in a relative decrease in 2,3-diphosphoglycerate. Stress also caused an increase in plasma corticosterone, which was negatively correlated with Leuconostocaceae. A reduction in 2,3-diphosphoglycerate would be detrimental to the organism since it is crucial for controlling the affinity of oxygen and hemoglobin (Benesch, Benesch & Yu, 1968).

## Conclusion

In conclusion, the results of 16S rRNA sequencing and GC-MS metabolomic analysis suggest that prenatal chronic stress not only causes negative emotions, but also alters the composition of the maternal gut flora and its metabolites involved in various metabolic pathways. Furthermore, these regulated metabolites associated with the gut flora may be potential biomarkers for further maternal damage. Therefore, we need to further investigate the causal relationship and model mechanisms between chronic stress during pregnancy and gut flora.

The effects of persistent, moderate, unexpected stress during pregnancy on the intestinal flora of SD rats were examined in this study. To find out if pregnant women who experience pregnancy-related anxiety have their gut flora affected, it is necessary to further research the effects of pregnancy anxiety on gut flora. Currently, samples of feces from pregnant women are being collected for our investigation. Additionally, this study solely looked at the gut microbiota of pregnant rats; the offspring were not given any particular characteristics. The effect on the gut flora of their offspring could be further investigated in subsequent experimental studies.

##  Supplemental Information

10.7717/peerj.15113/supp-1 Supplemental Information 1 Metabonomics

10.7717/peerj.15113/supp-2 Supplemental Information 2 Pregnant rat plasma corticosterone, elevated puzzle palace model and sugar water preference experiment

10.7717/peerj.15113/supp-3 Supplemental Information 3 Author Checklist - Full

10.7717/peerj.15113/supp-4 Supplemental Information 4 Alpha-diversity index of the intestinal microbiota of pregnant rats

## Additional Information and Declarations

### DNA Deposition

The following information was supplied regarding the deposition of DNA sequences: The raw reads are available at the NCBI Sequence Read Archive (SRA): PRJNA721070.

### Data Availability

The following information was supplied regarding data availability: The raw measurements are available in the Supplemental Files.
